# Supplementary material for: Red‐shifted channelrhodopsin stimulation restores light responses in blind mice, macaque retina, and human retina
Source: EMBO Mol Med. 2016 Sep 27;8(11):1248–64. doi: 10.15252/emmm.201505699 (PMC5090658; doi:10.15252/emmm.201505699)
Supplement: Supplementary file 1 — Expanded View Figures PDF [file EMMM-8-1248-s001.pdf]

Expanded View Figures

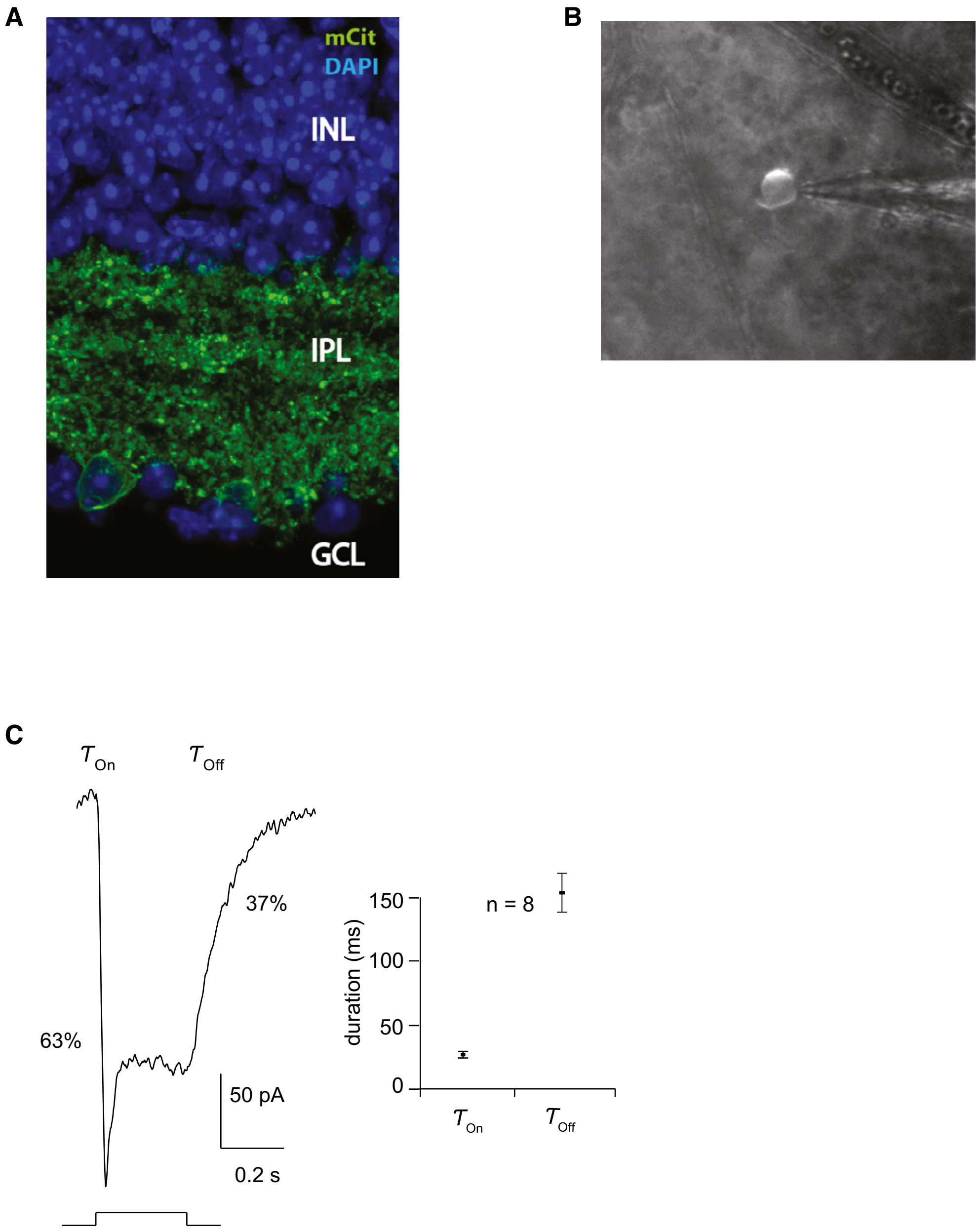

Figure EV1. Membrane/dendrite-targeted expression of ReaChR and electrophysiological properties of ReaChR-expressing RGCs in treated *rd1* mice.

A Vertical section through an AAV2-hSyn:ReaChR-mCitrine-injected *rd1* retina expressing mCitrine endogenous fluorescence localized to RGC membranes as well as RGC dendritic processes in the IPL. The nuclear stain DAPI was applied to label the retinal layers, inner nuclear layer (INL), inner plexiform layer (IPL), and ganglion cell layer (GCL). mCit was not amplified. Scale bar, 10 μm.

B Example of a mCitrine-positive RGC being approached with a patch pipette in a flat-mounted mouse retina, scale bar, 10 μm.

C On and off time constants (τ) calculated from a ReaChR photocurrent response; 1.2×10^{16} photons cm^{-2} s^{-1} at 550 nm (left) and results obtained from 8 cells (right). τ_{On} = 26 ± 2 ms, τ_{Off} = 153 ± 15 ms (mean ± SEM). Marks indicate the time needed to reach 63% of the onset peak value, and the decay time needed to reach 37% of the offset maxima (τ = 1).

 © 2016 The Authors

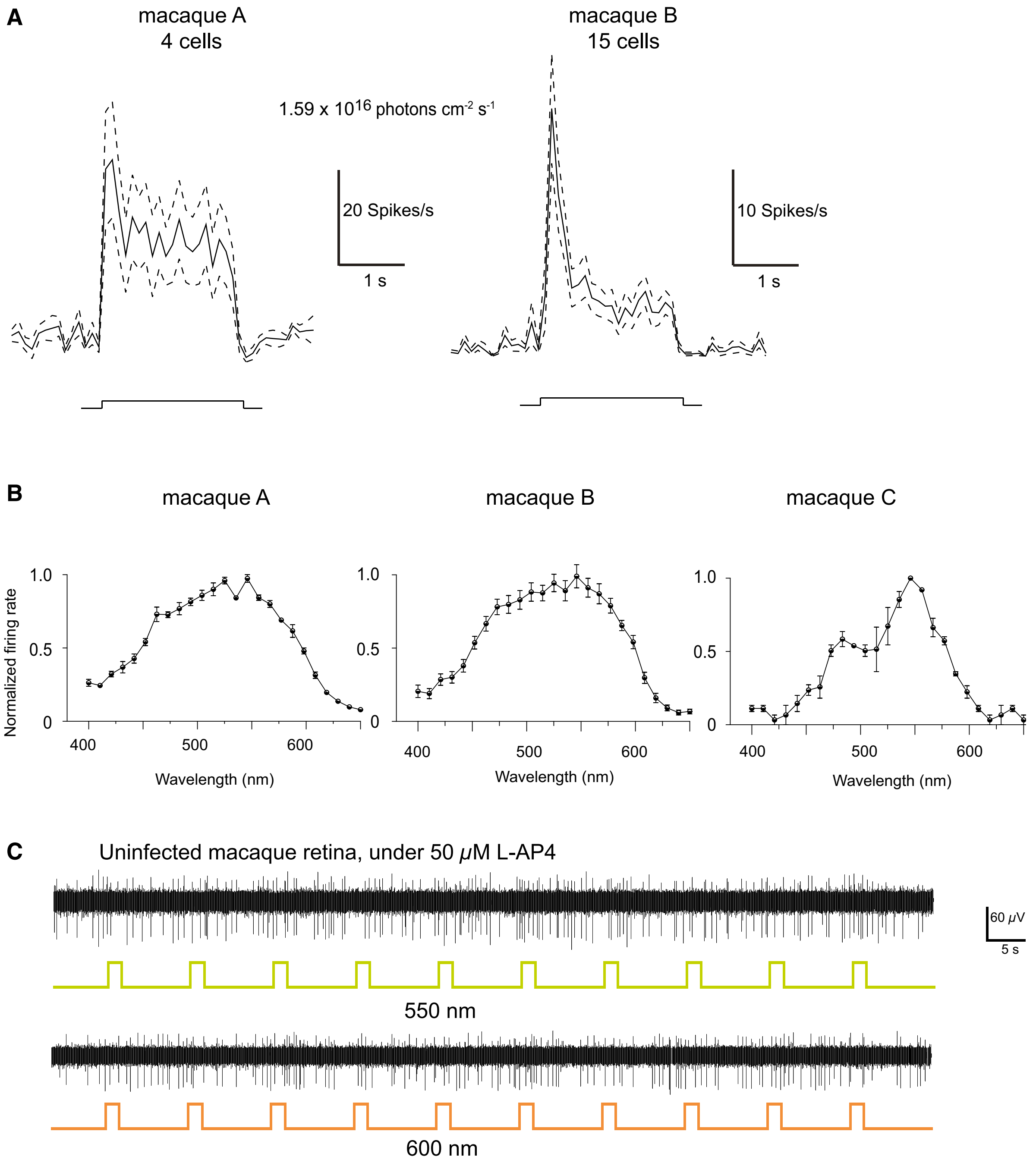

Figure EV2. ReaChR spike trains represented as peristimulus time histograms in two macaque retinae and individual ReaChR action spectrums of three macaque retinae treated with an AAV8 Y447 733F-CAG:ReaChR-GFP.

A Peristimulus time histograms (PSTH) of 4 cells from macaque A and 15 cells from macaque B built from 10 repetitions of a 2-s full-field stimulation at 1.59×10^{16} photons cm^{-2} s^{-1} (performed in AMES supplemented with L-AP4) and at 550 nm. The PSTH is presented as mean (solid line) and SEM (dashed lines). The light intensity used was below the safety threshold for light at 550 nm.

B Normalized action spectrums displayed individually for macaques A, B, and C (performed in AMES supplemented with L-AP4); each spectrum exhibits red-shifted peak RGC firing rates (peak = ~550 nm), similar to what was observed in ReaChR-treated *rd1* mice. Each plot was constructed by averaging the responses of all light-responding channels of a given macaque retinal explant, expressing ReaChR. Error bars represent standard error of means calculated over light-responding channels. Each plot represents an individual macaque's response, i.e. macaque A, B or C.

C Voltage trace of repetitions of 2-s full-field stimulation at 600 nm light (1.1×10^{17} photons cm^{-2} s^{-1}) and 550 nm light (at 1.59×10^{16} photons cm^{-2} s^{-1}) of an uninfected macaque retinal explant cultured *ex vivo* for 4 days (same duration as AAV incubation period), performed in AMES supplemented with L-AP4 demonstrates complete blockade of intrinsic light responses at light onsets, spontaneous uncorrelated spiking activity is still present.

Data information: All of the plots were obtained from multi-electrode array recordings performed in explanted macaque retinae.

© 2016 The Authors

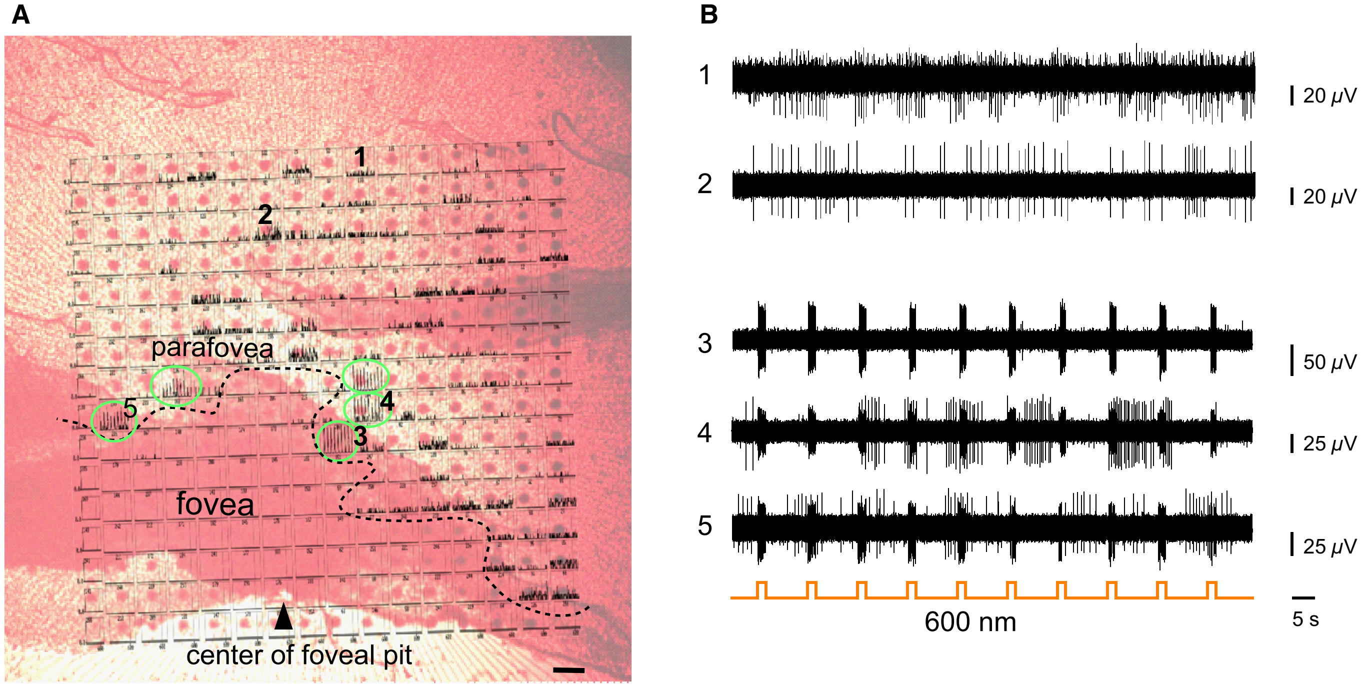

Figure EV3. The "electrophysiological boundary" of the fovea/para-fovea in the human macular explant.

A Infrared screen grab of the human macular explant piece pressed against the multi-electrode array captured during a recording session in which ReaChR-evoked light responses were observed. Superposed on the infrared picture of the retina is a representation of the firing rates recorded during that session for each of the 252 channels, obtained as a screenshot from the MC_Rack display (each rectangular window corresponds to the location of the recording electrode on the retina). The RGC-derived spike trains fall off at the boundary of the fovea. The black dashed line indicates the "electrophysiological boundary" of the para-fovea beyond which spontaneous spike trains were absent in the RGC-free fovea. ReaChR-evoked responses were observed in channels (highlighted in green), situated in close apposition to this boundary in the para-fovea. Scale bar, 100 μm (based on multi-electrode array electrode density).

B Voltage traces of representative recording electrodes from the multi-electrode array during the same recording. Traces 1 and 2 were obtained from electrodes that exhibited spontaneous activity only, but no light response; they are representative of the large number of electrodes in the para-fovea that exhibited spike trains in the explant, indicating that the retinal ganglion cells were viable and functional at the time of the experiment. Traces 3, 4, and 5 are from electrodes that exhibited ReaChR-evoked light responses (under LAP-4) in response to full-field 600-nm stimulation (1.1×10^{17} photons cm^{-2} s^{-1}) close to the fovea/para-foveal "electrophysiological boundary". The locations of the recording electrodes for traces 1–5 are indicated on the multi-electrode array image in (A).

 © 2016 The Authors

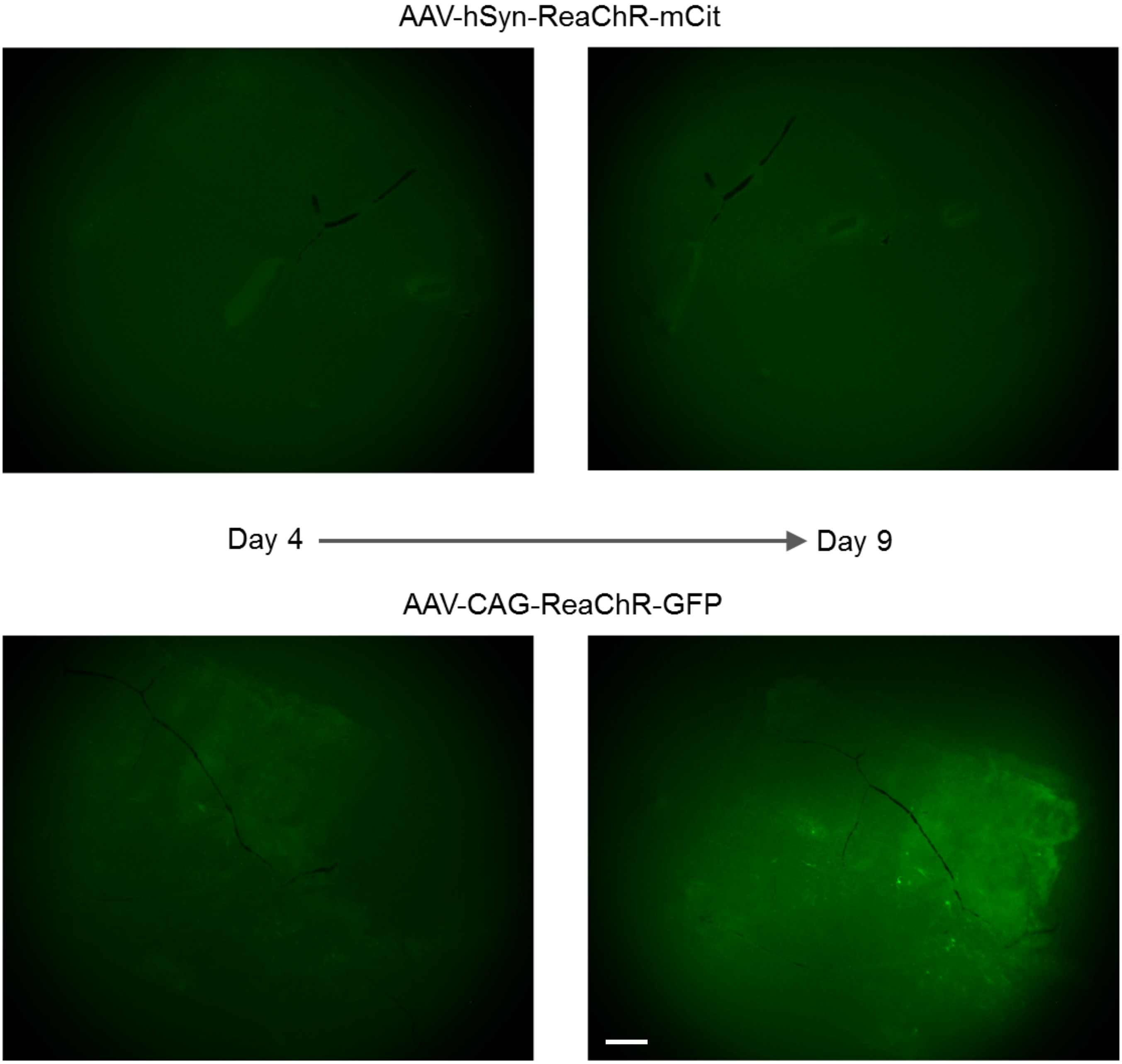

Figure EV4. Time course of AAV-mediated ReaChR expression in the human retina under cell culture.
Human retinal explants prepared from the far peripheral part infected with AAV2-hSyn:ReaChR-mCit (2.5×10^{13} vector genomes ml^{-1}) and AAV8 Y447 733F-CAG:ReaChR-GFP (6.3×10^{13} vector genomes ml^{-1}). After 9 days of viral infection, the time course shows very weak expression of the hSyn construct but a higher level of expression of the CAG construct in sparse populations of RGCs. Scale bar, 500 μm.

© 2016 The Authors
